# Heterogeneity of Intrinsic and Synaptic Properties of Neurons in the Ventral and Dorsal Parts of the Ventral Nucleus of the Lateral Lemniscus

**DOI:** 10.3389/fncir.2015.00074

**Published:** 2015-11-18

**Authors:** Franziska Caspari, Veronika J. Baumann, Elisabet Garcia-Pino, Ursula Koch

**Affiliations:** Neurophysiology, Institute of Biology, Freie Universität BerlinBerlin, Germany

**Keywords:** hearing, auditory brainstem, membrane properties, synaptic transmission, hyperpolarization-activated current

## Abstract

The ventral nucleus of the lateral lemniscus (VNLL) provides a major inhibitory projection to the inferior colliculus (IC). Neurons in the VNLL respond with various firing patterns and different temporal precision to acoustic stimulation. The present study investigates the underlying intrinsic and synaptic properties of various cell types in different regions of the VNLL, using *in vitro* electrophysiological recordings from acute brain slices of mice and immunohistochemistry. We show that the biophysical membrane properties and excitatory input characteristics differed between dorsal and ventral VNLL neurons. Neurons in the ventral VNLL displayed an onset-type firing pattern and little hyperpolarization-activated current (I_h_). Stimulation of lemniscal inputs evoked a large all-or-none excitatory response similar to Calyx of Held synapses in neurons in the lateral part of the ventral VNLL. Neurons that were located within the fiber tract of the lateral lemniscus, received several and weak excitatory input fibers. In the dorsal VNLL onset-type and sustained firing neurons were intermingled. These neurons showed large I_h_ and were strongly immunopositive for the hyperpolarization-activated cyclic nucleotide-gated channel 1 (HCN1) subunit. Both neuron types received several excitatory inputs that were weaker and slower compared to ventrolateral VNLL neurons. Using a mouse model that expresses channelrhodopsin under the promotor of the vesicular GABA transporter (VGAT) suggests that dorsal and ventral neurons were inhibitory since they were all depolarized by light stimulation. The diverse membrane and input properties in dorsal and ventral VNLL neurons suggest differential roles of these neurons for sound processing.

## Introduction

The ventral nucleus of the lateral lemniscus (VNLL), an auditory brainstem nucleus of the ascending auditory pathways, might play a critical role in the analysis of temporal sound patterns (Covey and Casseday, 1991; Recio-Spinoso and Joris, 2014). Neurons in the VNLL receive glutamatergic, excitatory inputs from several neuron types of the contralateral ventral cochlear nucleus (VCN) and a major glycinergic, inhibitory input from the ipsilateral medial nucleus of the trapezoid body (MNTB; Glendenning et al., 1981; Schofield and Cant, 1997; Irfan et al., 2005; Kelly et al., 2009). Most VNLL neurons are immunopositive for glycine and/or GABA and send inhibitory projections to the ipsilateral inferior colliculus (IC; Saint Marie et al., 1997; Riquelme et al., 2001; Zhang and Kelly, 2006). Based on the large number of GABAergic and glycinergic neurons in the VNLL, this nucleus represents a prominent inhibitory source to the IC (Saint Marie and Baker, 1990).

Neurons in the VNLL show diverse responses to sounds regarding temporal response patterns and binaurality (Batra and Fitzpatrick, 1997; Nayagam et al., 2006; Zhang and Kelly, 2006; Recio-Spinoso and Joris, 2014). Studies on brain slices have revealed various firing patterns in response to depolarizing current injections (Wu, 1999; Zhao and Wu, 2001; Irfan et al., 2005) that resemble firing pattern of VNLL neurons *in vivo* (Zhang and Kelly, 2006). A subset of VNLL neurons specifically responds to the onset of a sound with very short latencies and extremely low jitter (Covey and Casseday, 1991; Batra and Fitzpatrick, 1999; Zhang and Kelly, 2006). Anatomical studies suggest that these VNLL neurons receive a major excitatory input that arises from the octopus cells of the contralateral VCN (Adams, 1997; Schofield and Cant, 1997). These projections contact ventral VNLL neurons with calyx-like synapses that mediate information with high temporal fidelity (Berger et al., 2014).

So far the intrinsic and synaptic properties of VNLL neurons and their spatial distribution within the VNLL have not been systematically studied in animals when neuronal properties are considered to be mature (Khurana et al., 2012). To identify whether different neuron types are systematically distributed within the VNLL, as shown for echo-locating bats (Vater and Feng, 1990; Huffman and Covey, 1995), we characterized the intrinsic and synaptic properties of VNLL neurons in acute brain slices of young adult mice, relative to their location within the VNLL. Here, we show that the VNLL is a heterogeneous nucleus with neurons in the ventral and dorsal parts of the VNLL differing from each other in terms of their membrane properties, hyperpolarization-activated cyclic nucleotide gated (HCN) current density and their synaptic input characteristics. In the ventrolateral part of the VNLL a subpopulation of neurons with fairly uniform membrane and synaptic properties exists. These neurons receive one large excitatory synaptic input resembling Calyx of Held synaptic properties in the MNTB.

## Materials and Methods

### Animals

All experiments followed EU ethical guidelines and were carried out in accordance with protocols approved by the German federal authorities (Landesamt für Gesundheit und Soziales, State of Berlin).

All patch-clamp recordings and immunohistochemical labeling were performed in brain slices containing the VNLL of C57/Bl6J mice at the age of postnatal day 22/23 (denoted as P22) except for the experiments illustrated in Figure 7. For these experiments VGAT-ChR2-YFP^+^ [B6.Cg-Tg(Slc32a1-COP4*H134R/EYFP)8Gfng/J; The Jackson Laboratory] mice at P22 were used to determine inhibitory regions within the lateral lemniscus as channelrhodopsin-associated YFP is only expressed in vesicular GABA transporter (VGAT)-expressing neurons, hence GABAergic or glycinergic neurons (Zhao et al., 2011).

### Slice Preparation

The animals were decapitated under isoflurane anesthesia. The brains were removed and sliced in ice-cold oxygenated (95% O_2_/5% CO_2_) sucrose replacement solution containing (in mM): 2.5 KCl, 26 NaHCO_3_, 1.25 NaH_2_PO_4_, 6 MgCl_2_, 0.5 CaCl_2_, 25 glucose, 200 sucrose (pH 7.4). Transverse brainstem slices (180 μm) including the VNLL were cut with a vibratome (VT1200S; Leica, Germany), collected and stored at 32°C for 15 min for recovery in oxygenated artificial cerebrospinal fluid (ACSF) containing (in mM): 125 NaCl, 2.5 KCl, 1.25 NaH_2_PO_4_, 26 NaHCO_3_, 1 MgCl_2_, 2 CaCl_2_, 25 glucose and then kept at room temperature until recording for up to 4–5 h. For patch clamp experiments, slices were transferred to a recording chamber, which was perfused continuously with oxygenated ACSF at near-physiological temperature of 32°C. An upright microscope (Axioscope, Zeiss, Germany) with infrared-differential interference contrast optics was attached to visualize the neurons.

### Electrophysiology

**Figure 1:**
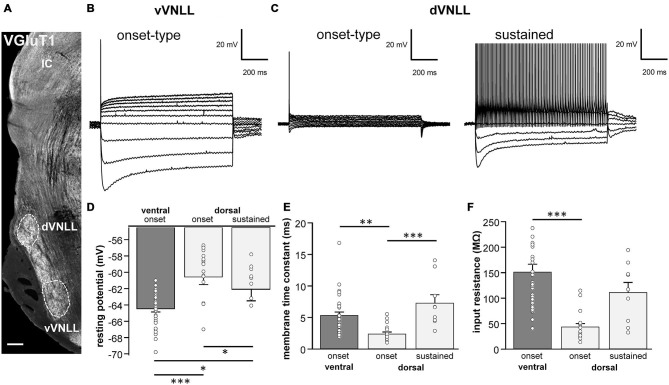
**Intrinsic properties differ between vVNLL and dVNLL neurons. (A)** Low-power image with VGluT1-labeling illustrates the relative location of the VNLL within the auditory brainstem. The dorsal and ventral parts of the VNLL (dVNLL and vVNLL, respectively) are marked with a dashed line to show the respective recording sites. Scale bar: 200 μm. **(B)** Representative voltage responses to hyperpolarizing and depolarizing current injections recorded from vVNLL neurons. Depolarizing current injections elicited an onset-type firing pattern in vVNLL neurons. **(C)** Depolarizing current injections elicited different types of firing patterns in dVNLL neurons: onset-type (left) and sustained (right) firing pattern. **(D)** Resting potential, **(E)** membrane time constant and **(F)** peak input resistance for vVNLL and dVNLL neurons. dVNLL: onset-type *n* = 19, sustained *n* = 9, vVNLL: onset-type *n* = 34. Data are obtained from twelve C57/Bl6J mice. Statistical significance was determined by a single-factor ANOVA test followed by a Scheffé’s *post hoc* test; *p < 0.05, **p < 0.01, ***p < 0.001.

Whole-cell patch-clamp recordings were made from visually identified VNLL neurons using a Multiclamp 700 A amplifier (Axon Instruments, USA). Patch pipettes were pulled from borosilicate glass capillaries (BioMedical Instruments, Germany, 0.86 mm inner diameter and 1.5 mm outer diameter) on a DMZ Universal Puller (Zeitz Instruments, Germany) and filled with the following solutions: for current clamp recordings and hyperpolarization-activated current (I_h_) measurements: (in mM) 125 K-gluconate, 5 KCl, 10 HEPES, 1 EGTA, 2 Na_2_ATP, 2 MgATP, 0.3 Na_2_GTP, 10 Na-phosphocreatinine (pH 7.25); for recordings of synaptic currents: 41 CsCl, 99 Cs-methylsulfonate, 10 HEPES, 10 Cs-EGTA, 2 Na_2_ATP, 2 MgATP, 0.3 Na_2_GTP, 1 CaCl_2_, 5 TEA-Cl, 5 QX 314 (pH 7.4). All experiments were performed at 32°C. Patch pipettes had resistances between 2–4 MΩ. To study the intrinsic membrane properties with respect to their location within the VNLL, the VNLL was subdivided into three regions: dorsal, middle and ventral based on the nomenclature proposed by Benson and Cant (2008) for gerbils. We mainly recorded from the ventral and dorsal parts of the VNLL (vVNLL and dVNLL, respectively; Figure 1A). The ventral VNLL was considered as the ventral end of the fiber bundle of the lateral lemniscus and the dorsal VNLL was determined with respect to the dorsal nucleus of the lateral lemniscus (DNLL). The neurons location was determined in the acute slice preparation by anatomical landmarks and delineated in a sketch of the VNLL. In some experiments Alexa 555 was added to the internal solution in order to later verify the visually determined location of a neuron within the VNLL.

Bridge balance was adjusted for current-clamp recordings. During voltage-clamp recordings series resistance (<10 MΩ) was compensated to a residual resistance of 2–2.5 MΩ and was not allowed to change more than 20% during the recording. For measuring passive and active membrane properties 1000 ms long hyperpolarizing and depolarizing current steps from −300 pA to 1000 pA were injected. Channelrhodopsin was activated with a 5–10 ms light pulse of the Arc lamp which yields similar depolarization amplitude of VGAT-positive neurons as a 26.3 mW/mm^2^ blue LED (Zhao et al., 2011).

According to Baumann et al. (2013) HCN-currents were isolated by pharmacological blockade of other ionic currents by applying the following drugs to the bath (in mM): 1 3, 4-diaminopyridine, 10 TEA-Cl, 0.2 BaCl_2_, 0.001 TTX, 0.05 NiCl_2_, 0.1 CdCl_2_, 0.01 DNQX, 0.025 DL-AP5 and 0.001 strychnine. The NaCl concentration was reduced to maintain iso-osmolarity. I_h_ was activated by applying depolarizing and hyperpolarizing voltage steps from −40.5 to −120.5 mV. Tail currents were measured at a holding potential of −100.5 mV that followed each voltage step. Current densities were obtained by estimating cell surface size from the capacitance compensation measurements of the amplifier.

Synaptic currents were evoked by stimulating the ascending fibers 50 μm ventral to the recorded neuron with a glass electrode filled with 2 M NaCl. Stimuli consisted of brief biphasic pulses (200 μs, intensities 5–80 V) triggered by an analog stimulus isolation unit (BSI-950, Dagan Corporation, USA). Stimulation intensities between 5–80 V were used in these experiments. Threshold for synaptic responses was usually between 10–20 V. For the analysis of input number the stimulation intensity was gradually increased in increments of 2–10 V. Inhibitory (glycinergic and GABAergic) or excitatory (AMPA and NMDA) postsynaptic currents were isolated by applying 1 μM Strychnine and 10 μM SR95531 or 10 μM DNQX and 25 μM DL-AP5 to the bath, respectively.

All agents were purchased from Sigma-Aldrich (Seelze, Germany) and Biotrend (Köln, Germany) unless otherwise indicated.

### Data Acquisition and Analysis

For all electrophysiological measurements, stimulus generation and recordings were acquired using pCLAMP (Axon Instruments, USA). Current and voltage clamp signals were low-pass filtered at 10 kHz with a Bessel filter, sampled at a rate of 20–50 kHz and analyzed using Clampfit 10.0 (Axon Instruments, USA) and customized macros in IGOR Pro (Wavemetrics, USA). A junction potential of −10.5 mV was subtracted for recordings using a K-Gluconate solution.

The resting membrane potential was calculated as the mean value of the first 80 ms before hyperpolarizing or depolarizing steps were applied. Membrane time constant and input resistance were evaluated from the voltage response to a current injection of −100 pA. Membrane time constants were calculated by fitting single-exponential functions to the voltage traces. Input resistance was measured at the peak hyperpolarization according to Ohm’s law. For analyzing the active membrane properties, the action potential or the train of action potentials elicited by the lowest current injection was used to measure current threshold, voltage threshold, action potential amplitude, half-width, and latency.

Half-maximal activation of I_h_ was computed from tail currents. Tail current amplitudes were measured 20 ms after the termination of the hyperpolarizing voltage steps. These amplitudes were normalized to the maximal amplitude of each neuron. Values were fitted to a Boltzmann function to obtain the half-maximal activation voltage V_1/2_.

During synaptic stimulation experiments the number of input fibers was determined by visually identifying groups of similar excitatory post-synaptic current (EPSC) and inhibitory post-synaptic current (IPSC) amplitudes while stimulation intensities were gradually increased from 10–80 V. Each group presumably reflects the activation of an individual input fiber (Kim and Kandler, 2003; Walcher et al., 2011).

10−90% rise time and tau decay (fitting of a single-exponential function) of synaptic currents were assessed from responses evoked by minimal stimulation. The minimal stimulation strength is defined as stimulation strength where a stepwise increase of 2 V results in the first measureable post synaptic current (PSC). To estimate short-term plastic changes of excitatory and inhibitory inputs to repetitive stimulations (1–300 Hz, 15 pulses, 10 repetitions), PSC amplitudes in the trains were normalized to the first amplitude. For calculating net amplitudes at high frequencies, a decay function was fitted to the preceding current pulse and set as new baseline. The amplitude of the respective pulse was measured as a subtraction of the maximum and the new baseline. From these normalized depression curves steady state depression was obtained by the mean of the last three PSC amplitudes.

Results are shown as mean ± standard error of the mean. Statistical significance was either calculated by the unpaired student’s *t*-test or single factor ANOVA followed by a Scheffé’s *post hoc* test in Excel (Microsoft) or Igor Pro (Wavemetrics, USA) with statistical thresholds of *p < 0.0, **p < 0.01 and ***p < 0.001. Kruskal-Wallis test was used, as indicated, for non-parametric distributions.

### Immunohistochemistry

Animals were anesthetized by an intraperitoneal injection of Fentanyl (Janssen-Cilag), Medetomidin (Ratiopharm) and Metformin (Pfizer). They were perfused transcardially with 0.1 M phosphate buffer (PB; pH 7.4) for 3 min followed by Paraformaldehyde (Carl Roth, Karlsruhe, Germany; PFA; 4% in 0, 1 M PB; pH 7.4) for 15 min. Immediately after perfusion, brains were removed and post-fixed overnight in 4% PFA at 4°C. Brains were thoroughly washed at room temperature with 0.1 M phosphate buffered saline (PBS; pH 7.4). Coronal brain sections of 50 μm were obtained using a vibratome (VT1200, Leica, Germany). Sections containing the VNLL were collected. Unspecific binding was blocked incubating the sections in solution containing 10% normal donkey serum (NDS; GeneTex, USA), 0.2% Triton X-100 (Carl Roth, Karlsruhe, Germany) and 0.1 M PBS, for an hour at room temperature. Slices were subsequently incubated overnight in the primary antibody sera containing mouse α-HCN1 (NeuroMab; dilution 1:1000), rabbit α-VGluT1 (Synaptic Systems; dilution 1:500), chicken α-MAP2 (Neuromics; dilution 1:1000), goat α-Calretinin (Millipore; dilution 1:4000), 3% NDS, and 0.2% Triton X-100, in 0.1 M PBS.

Slices were washed in 0.1 M PBS and incubated for 2 h at room temperature in secondary antibody sera containing 3% NDS, 0.2% Triton X-100, Alexa 488 donkey α-mouse (life technologies; dil. 1:250), Alexa 555 donkey α-rabbit (life technologies; dilution 1:500), Cy3 donkey α-goat (Dianova; dilution 1:300), and Alexa 647 donkey α-chicken (Dianova; dilution 1:300). Negative controls were obtained by omitting the primary antibody. Sections were washed several times in 0.1 M PBS, mounted and covered with homemade anti-fading mounting media (Indig et al., 1997). All sections were analyzed with a Leica SP8 confocal laser scanning microscope. Three laser lines for visualization were used. The fluorochromes Alexa 488, Alexa 555 and Alexa 647 were excited by an Ar-Ion-Laser line of 488 nm, the single DPSS 561 nm laser line and the HeNe single laser line of 633 nm, respectively. Stacks of confocal images were further analyzed and edited with ImageJ (NIH, USA).

## Results

### Intrinsic Properties Differ between Neurons of the Dorsal and the Ventral Parts of the VNLL

Neuron types are largely determined by their biophysical membrane properties which are dependent on the composition of ion channels in the membranes. To find out whether there is a systematic distribution of neuronal cell types across the VNLL we analyzed intrinsic membrane properties with respect to their location within the VNLL in a brain slice preparation of young adult mice (P22/23).

The VNLL is an elongated structure with a ventral and a dorsal area that are enriched with neurons (Figure 1A). In ventral VNLL (vVNLL) neurons, depolarizing current injections elicited an onset-type firing pattern in 34 out of 37 (Figure 1B). In the remaining three neurons depolarization induced an onset burst firing pattern (data not shown). In all vVNLL neurons, a negative current injection led to a large hyperpolarization followed by a slowly depolarizing voltage sag. In the dorsal part of the VNLL (dVNLL), positive current injections induced either an onset-type firing pattern (*n* = 19) or a sustained firing pattern (*n* = 9; Figure 1C). In onset-type firing neurons negative current injections induced a small hyperpolarization which repolarized rapidly. In sustained-firing neurons, the hyperpolarization was more pronounced with a prominent voltage sag. Onset-type firing neurons of the vVNLL and dVNLL significantly differed in their resting membrane potential, their membrane time constant and their input resistance (Figures 1D–F; Table 1). Onset-type firing neurons of the dVNLL were fastest and exhibited the smallest peak input resistance suggesting different subpopulation of onset-type firing neurons in the vVNLL and dVNLL.

**Table 1:** **Summary of membrane properties of dVNLL and vVNLL neurons**.

	Ventral	Dorsal		
	Onset (*n* = 34)	Onset (*n* = 19)	Sustained (*n* = 9)	*p*
**Passive membrane properties**				
Resting potential (mV)	−64.5 ± 0.4	−60.6 ± 0.9	−62.1 ± 1.4	*^a, b,^ ***^c^
Input resistance (MΩ)	151.7 ± 43.8	43.8 ± 6.3	111.4 ± 19.4	***^c^
Membrane time constant (ms)	5.36 ± 0.51	2.41 ± 0.29	7.31 ± 1.30	**^c,^ ***^a^
**Active membrane properties**				
Current threshold (pA)	376.5 ± 19.4	500.0 ± 33.3	266.7 ± 50.0	*^c,^ ***^a^
Voltage threshold (mV)	−36.7 ± 0.6	−33.5 ± 1.2	−34.5 ± 1.1	*^c^
AP amplitude (mV)	58.7 ± 0.9	56.6 ± 2.0	66.7 ± 2.6	*^b,^ **^a^
AP half-width (ms)	0.47 ± 0.03	0.46 ± 0.03	0.39 ± 0.02	n.s
AP latency (ms)	2.0 ± 0.1	2.5 ± 0.3	4.0 ± 0.4	**^a,^ ***^b^

Values are mean ± SEM. The level of significance between ventral and dorsal was determined using Scheffé’s post hoc test following a single-factor ANOVA test (*p < 0.05, **p < 0.01, ***p < 0.001). ^a^Significant differences between dVNLL onset-type and dVNLL sustained firing neurons. ^b^Significant differences between dVNLL sustained firing and vVNLL neurons. ^c^Significant differences between vVNLL and dVNLL onset-type firing neurons.

**Figure 2:**
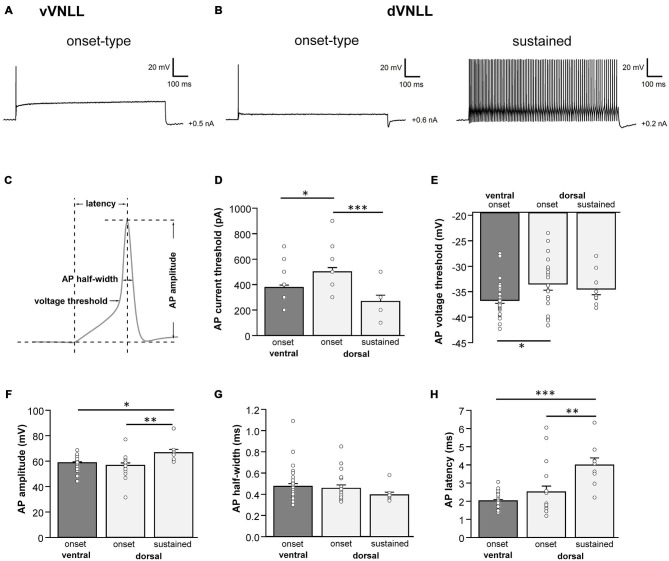
**Heterogeneous firing properties differ between neurons of the dVNLL and vVNLL. (A)** Representative voltage traces recorded from a vVNLL neuron **(A)** and from dVNLL neurons **(B)** during a depolarizing current injection. **(C)** Schematic diagram illustrating the analysis of the active membrane properties. The first action potential (AP) elicited by the lowest current injection was used for analysis. **(D)** Current threshold eliciting the first action potential for vVNLL and dVNLL neurons. **(E)** Voltage threshold, **(F)** AP amplitude, **(G)** AP half-width and **(H)** AP latency of the first elicited AP for vVNLL and dVNLL neurons. dVNLL: onset-type *n* = 19, sustained *n* = 9, vVNLL onset-type *n* = 34. Statistical significance was determined by a single-factor ANOVA test followed by a Scheffé’s *post hoc* test; *p < 0.05; **p < 0.01; ***p < 0.001.

We next analyzed active membrane properties of the different VNLL cell types at the first supra-threshold response (Figures 2A,B). Onset-firing neurons of the ventral and the dorsal regions differed significantly in current and voltage threshold, whereas sustained firing neurons of the dorsal part displayed differences to onset-type firing neurons of both regions regarding AP amplitude and AP latency (Figures 2C–H; Table 1). Sustained-firing neurons of the dVNLL had the lowest current threshold, largest AP amplitudes and latencies, whereas onset-type firing cells of the same region had the highest current threshold of all neurons measured (Figures 2D,F,H; Table 1). AP half-width did not differ between vVNLL and dVNLL neurons (Figure 2G; Table 1).

We conclude that the dVNLL consists of two different subpopulations of neurons with distinct intrinsic membrane properties. The population of vVNLL neurons, however, seems to be fairly uniform regarding their intrinsic properties.

### Dorsal VNLL Neurons have Large Hyperpolarization-Activated Currents (I_h_) Compared to Ventral VNLL Neurons

**Figure 3:**
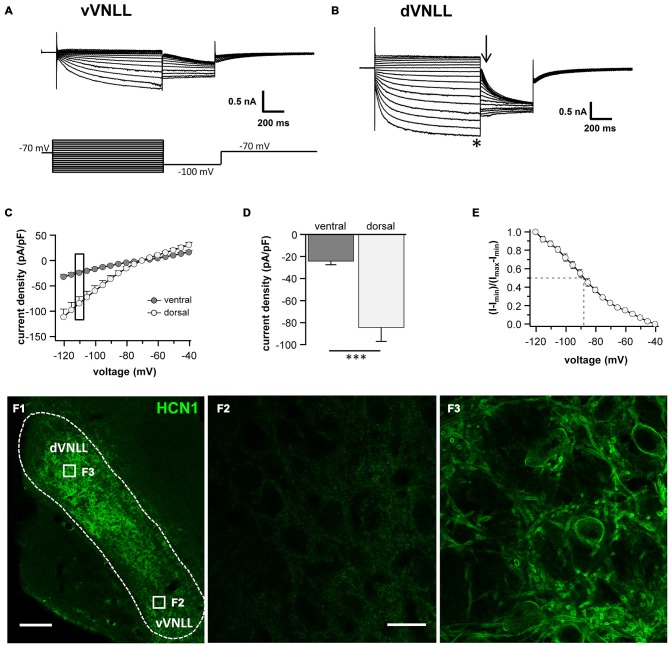
**DVNLL neurons have larger hyperpolarization-activated currents (I_h_) and stronger HCN1 immunoreactivity.** Representative current traces recorded from vVNLL neurons **(A)** and dVNLL neurons **(B)** to voltage steps ranging from −40 to −120 mV in 5 mV step increment. Each voltage step lasted for 1 s and was followed by a voltage step to −100 mV for 0.5 s to elicit the tail current to determine the voltage dependence of I_h_ activation. Holding potential was set to −70 mV. **(C)** Current density-voltage relationship for dVNLL and vVNLL neurons. Current amplitudes were measured at steady-state (indicated by the asterisk in **B**). Rectangular labeling is exemplified in **(D)**. **(D)** Current density at −110 mV for vVNLL and dVNLL neurons are significantly different. dVNLL: *n* = 12, vVNLL *n* = 13. Statistical significance was determined by an unpaired student’s *t*-test; ***p < 0.001. **(E)** The voltage-dependence of I_h_ activation was determined from the tail current 20 ms after the end of the voltage steps (indicated by the arrow in **B**). Values were fitted with a Boltzmann function. Half-maximal activation voltage for neurons was at around −88 mV (*n* = 10). Half-maximal activation voltage of vVNLL neurons is not shown since almost no current is present. Data are obtained from eight C57/Bl6J mice. **(F)** Confocal images of HCN1 immunostaining in the VNLL. **(F1)** Overview illustrating a gradient of HCN1 staining in the VNLL. **(F2)** High magnification of a region within the vVNLL reveals that HCN1 staining is almost absent. **(F3)** High magnification of a region within the dVNLL shows strong HCN1 staining confined to the membrane. Scale bar: **(F1)** 200 μm; **(F2)** 20 μm.

Differences in the resting membrane potential and in the voltage sag induced by hyperpolarization between dorsal and ventral VNLL neurons indicate a differential distribution of HCN channels between the two parts. Our electrophysiological measurements of I_h_ in different parts of the VNLL revealed only small I_h_ amplitudes in the vVNLL (Figure 3A), whereas I_h_ amplitudes were large in dVNLL neurons (Figure 3B). Consequently, I_h_ density was significantly larger in dVNLL neurons as compared to vVNLL neurons (at −110.5 mV vVNLL: −24.0 ± 3.4 pA/pF, *n* = 13; dVNLL: −84.3 ± 12.9 pA/pF, *n* = 12; student’s unpaired *t*-test, *p* = 0.0006; Figures 3C,D). Half-maximal activation voltage for HCN channels in the dVNLL was −88.3 ± 3.0 mV (*n* = 10, Figure 3E), whereas tail-currents evoked in vVNLL neurons were too small to be reliably analyzed. Consistently, immunostainings against the HCN1 subunit showed that this subunit is strongly expressed in the dVNLL, whereas neurons in the vVNLL are mostly HCN1 negative (Figures 3F1–F3) indicating that HCN1 channels are the predominant isoform in dVNLL neurons.

Neurons in the vVNLL all have onset-type firing properties, little I_h_ and relatively large input resistance during hyperpolarization. In contrast, all dVNLL neurons have large I_h_ currents and small input resistance which results in short membrane time constants during hyperpolarization.

### Lateral vVNLL Neurons have Extremely Large Excitatory Postsynaptic Currents

**Figure 4:**
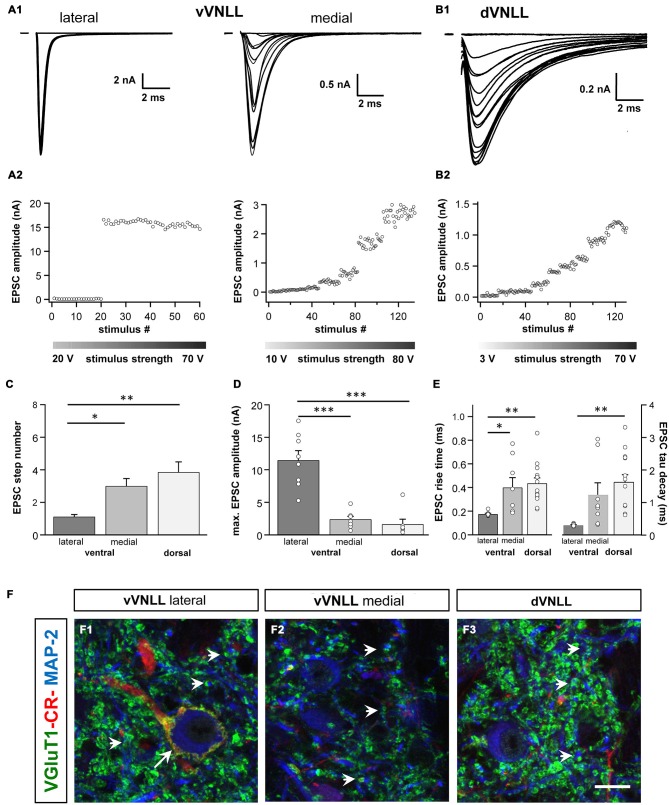
**Excitatory postsynaptic currents (EPSCs) are larger and the excitatory inputs are stronger in vVNLL neurons. (A1)** Representative EPSC traces of ventrolateral (left) and ventromedial (right) VNLL neurons. Responses were evoked by increasing stimulation strength. One strong input was found in most ventrolateral neurons, whereas medial neurons showed multiple inputs. All traces represent the average of at least three consecutive recordings to the same stimulus strength. **(A2)** EPSC amplitude as a function of stimulus number recorded from ventrolateral and ventromedial vVNLL neurons shown in **(A1)**. **(B1)** Representative EPSC traces of dVNLL neurons. Responses were evoked with increasing stimulation strength. This neuron shows seven distinct current steps. Note the considerably smaller maximal amplitude compared to ventrolateral VNLL neurons. All traces represent the average of at least three consecutive recordings to the same stimulus strength. **(B2)** EPSC amplitude as a function of stimulus number recorded from the dVNLL neuron shown in **(B1)**. **(C)** Number of EPSC steps evoked by increasing stimulation strength in vVNLL and dVNLL neurons. Ventrolateral VNLL neurons generally receive one, maximally two excitatory inputs whereas ventromedial and dVNLL neurons receive one to seven inputs. vVNLL: lateral *n* = 8, medial *n* = 8; dVNLL *n* = 7. **(D)** Maximal EPSC amplitude is significantly larger in ventrolateral vVNLL neurons. vVNLL: lateral *n* = 8, medial *n* = 8; dVNLL *n* = 7. **(E)** Rise time and tau decay time were measured in vVNLL and dVNLL neurons applying a minimal stimulation paradigm. Ventrolateral neurons are significantly faster than dVNLL neurons. vVNLL: lateral: *n* = 9; medial: *n* = 8; dVNLL: *n* = 14. Statistical significance was determined by a single-factor ANOVA test followed by a Scheffé’s *post hoc* test; *p < 0.05, **p < 0.01, ***p < 0.001. Data are obtained from 21 C57/Bl6J mice. **(F)** Confocal images depicting VGlut1, calretinin (CR) and MAP-2 immunofluorescence in the lateral vVNLL **(F1)**, the medial vVNLL **(F2)** and the dVNLL **(F3)**. Note a VGluT1 and Calretinin positive ring only found around lateral vVNLL neurons (**F1**: long arrow) whereas punctate VGluT1 staining in the neuropil is widely distributed along the VNLL **(F1–F3)**. Scale bar: 10 μm.

We were further interested to what extent the excitatory and inhibitory input properties differed between the two regions. Inputs were stimulated in the fiber bundle about 50 μm ventral to the recorded neuron and EPSCs were isolated pharmacologically. Stimulation intensity was systematically increased to estimate the number of input fibers (=step number; see “Materials and Methods” Section). During these experiments we noticed that the vVNLL can be subdivided into two neuronal population based on the estimated number and the peak amplitude of excitatory inputs the neurons received. A large population of vVNLL neurons that were located in a cluster laterally to the fiber bundle all exhibited an all-or-none EPSC response with extremely large amplitudes irrespective of stimulation strength (Figures 4A1,A2 left). This was different in the ventromedial part of the VNLL where neurons are scattered within the fiber bundle. There, EPSC amplitude gradually increased with rising stimulation strength which indicates a convergence of several input fibers (Figures 4A1,A2 right). Step number and maximally evoked EPSC amplitude differed significantly between ventrolateral and ventromedial VNLL neurons (Figures 4C,D; Table 2). Excitatory input properties of neurons in the dVNLL very much resembled those found in the medial neurons of the vVNLL (Figures 4B1,B2; Table 2). Moreover, 10–90% rise time and tau decay time of EPSCs were much faster in ventrolateral compared to ventromedial and dorsal neurons (Figure 4E; Table 2). We reanalyzed membrane properties according to their mediolateral location in the vVNLL. Nevertheless, membrane properties were very similar in medial and lateral VNLL neurons despite the profound differences in excitatory input characteristics (*resting membrane potential*: ventrolateral −64.4 ± 0.4 mV, *n* = 29, ventromedial −65.2 ± 0.8 mV, *n* = 5; *input resistance*: −150.17.3 MΩ, *n* = 29; ventromedial −158.8 ± 26.5 MΩ, *n* = 5; *membrane time constant*: ventrolateral 5.3 ± 0.58 ms, *n* = 29; ventromedial 5.71 ± 1.11 ms, *n* = 5, data not shown). These physiological findings were corroborated by a differential distribution of VGluT1 and calretinin positive presynaptic structures in different parts of the VNLL. Whereas in the ventrolateral VNLL a distinct perisomatic VGluT1-positive ring, that co-localized with calretinin, was detected around the somata (Figure 4F1), in both the ventromedial VNLL and the dorsal VNLL VGluT1-staining appeared scattered in the neuropil, presumably representing dendritic inputs (Figures 4F2,F3). This implies that neurons in the ventrolateral VNLL receive one large excitatory synapse with calyx-like properties similar to MNTB neurons. In contrast, medioventral and dorsal VNLL neurons integrate several excitatory inputs with smaller amplitudes that terminate predominantly on the dendrites.

**Table 2:** **Summary of synaptic properties of dVNLL and vVNLL neurons**.

	Ventral		Dorsal	
	Lateral	Medial		*p*
**Excitatory postsynaptic currents**				
Step number	1.13 ± 0.13 (8)	3.00 ± 0.46 (8)	3.85 ± 0.63 (7)	*^a,^ **^b^
Max. EPSC amplitude (nA)	11.47 ± 1.48 (8)	2.39 ± 0.45 (8)	1.66 ± 0.76 (7)	***^a, b^
10–90% rise time (ms)	0.18 ± 0.01 (9)	0.40 ± 0.08 (8)	0.44 ± 0.04 (14)	*^a,^ **^b^
Tau decay (ms)	0.31 ± 0.01 (9)	1.24 ± 0.37 (8)	1.63 ± 0.24 (14)	**^b^
**Inhibitory postsynaptic currents**				
Step number	2.70 ± 0.26 (10)	2.38 ± 0.26 (8)	2.58 ± 0.36 (12)	
Max. EPSC amplitude (nA)	5.95 ± 1.03 (10)	2.80 ± 1.08 (8)	2.80 ± 0.81 (12)	
10–90% rise time (ms)	0.37 ± 0.02 (16)	0.49 ± 0.07 (10)	0.48 ± 0.07 (14)	
Tau decay (ms)	2.47 ± 0.17 (16)	1.88 ± 0.11 (10)	3.06 ± 0.68 (14)	

Values are mean ± SEM. The level of significance between ventral and dorsal was determined using Scheffé’s post hoc test following a single-factor ANOVA test or Games Howell post hoc test following Kruskal-Wallis for non-parametric distributions (*p < 0.05, **p < 0.01, ***p < 0.001). Numbers of neurons are given in brackets. ^a^Significant differences between lateral and medial neurons of the vVNLL. ^b^Significant differences between dVNLL and lateral vVNLL neurons.

### Inhibitory Postsynaptic Currents are Similar in all vVNLL Neurons

**Figure 5:**
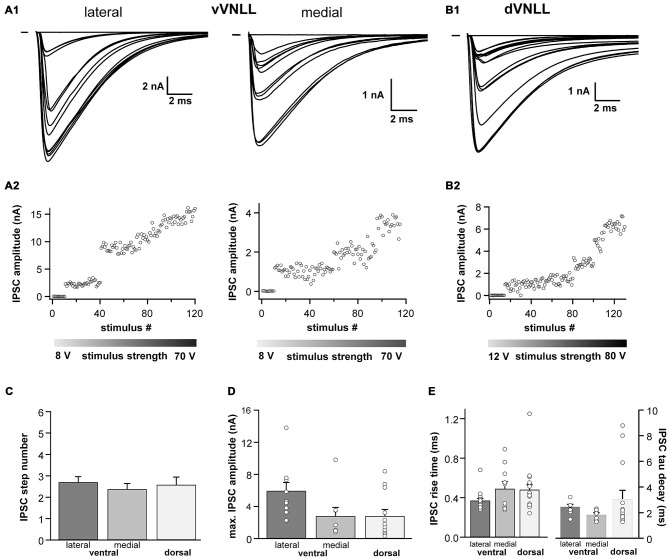
**Inhibitory inputs are similar in vVNLL and dVNLL neurons. (A1)** Representative IPSC traces of ventrolateral (left) and ventromedial (right) VNLL neurons. Responses were evoked by increasing stimulation strength. Multiple inputs were found both in ventrolateral and ventromedial neurons. All traces represent the average of at least three consecutive recordings to the same stimulus strength. **(A2)** IPSC amplitude as a function of stimulus number recorded from the ventrolateral and ventromedial vVNLL neurons shown in **(A1)**. **(B1)** Representative IPSC traces of dVNLL neurons. Responses were evoked by increasing stimulation strength. This neuron shows four distinct current steps. All traces represent the average of at least three consecutive recordings to the same stimulus strength. **(B2)** IPSC amplitude as a function of stimulus number recorded from the dVNLL neuron shown in **(B1)**. Multiple current steps can be seen. **(C)** Number of IPSC steps evoked by increasing stimulation strength in vVNLL and dVNLL neurons. VNLL neurons receive two to three inputs. vVNLL: lateral *n* = 10, medial *n* = 8; dVNLL *n* = 12. **(D)** Maximal IPSC amplitude is similar in all VNLL regions. vVNLL: lateral *n* = 10, medial *n* = 8; dVNLL *n* = 12. **(E)** Rise time and tau decay time were measured in vVNLL and dVNLL neurons applying a minimal stimulation paradigm. vVNLL: lateral: *n* = 16; medial: *n* = 10; dVNLL: *n* = 14. Data are obtained from 16 C57/Bl6J mice.

We were also interested whether inhibitory input characteristics differed between the various subregions of the VNLL (Figures 5A1,A2,B1,B2). In all three regions of the VNLL the number of inhibitory inputs was very similar (Figure 5C; Table 2). In accordance with the excitatory input strength, maximal inhibitory input amplitude was significantly larger in ventrolateral neurons compared to other VNLL areas (Figure 5D; Table 2). The temporal characteristics of inhibitory inputs were very similar in all parts of the VNLL (Figure 5E; Table 2).

Taken together, we show that VNLL neurons exhibit similar inhibitory synaptic responses which suggest a common origin of these inputs.

### Synaptic Short-Term Plasticity of Excitatory and Inhibitory Inputs is Similar in vVNLL and dVNLL Neurons

**Figure 6:**
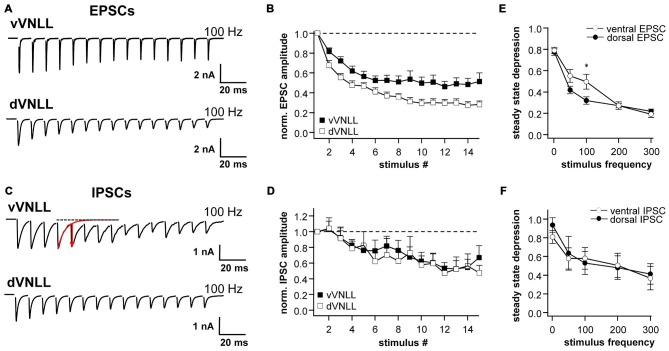
**Synaptic short-term plasticity of excitatory and inhibitory inputs is similar in vVNLL and dVNLL neurons.** Representative EPSC **(A)** and IPSC **(C)** traces in response to 15 stimulations at 100 Hz illustrating short-term plasticity in ventrolateral and dorsal VNLL neurons. Net amplitudes at high frequencies were calculated by fitting a decay function (red trace in **C**) to the preceding current pulse which was used to set as new baseline. The amplitude of the respective pulse was measured as a subtraction of the maximum and the new baseline (illustrated as red arrow in **C**). Normalized EPSC **(B)** and IPSC **(D)** amplitudes for 100 Hz stimulus in ventrolateral and dorsal VNLL neurons. Steady state depression of EPSCs **(E)** and IPSCs **(F)** in ventrolateral and dorsal VNLL neurons. Statistical significance was determined by an unpaired student’s *t*-test; *p < 0.05. IPSCs: dorsal: *n* = 9, ventral: *n* = 9; data are obtained from seven C57/Bl6J mice. EPSCs: dorsal: *n* = 12, ventral: *n* = 11; data are obtained from ten C57/Bl6J mice.

We further characterized short-term plasticity (STP) of excitatory and inhibitory synapses in vVNLL and dVNLL neurons (15 stimuli at 1, 50, 100, 200 and 300 Hz). All excitatory and inhibitory inputs to VNLL neurons showed pronounced synaptic short-term depression that depended on the stimulation frequency (Figures 6A–D). In order to compare synaptic short-term depression of vVNLL and dVNLL neurons the steady state depression as a function of stimulus frequency is shown in Figures 6E,F. For both EPSCs and IPSCs steady state depression did not differ between vVNLL and dVNLL neurons for almost all stimulus frequencies. Only for stimulation frequencies of 100 Hz steady state depression of EPSCs was significantly larger for vVNLL compared to dVNLL neurons (vVNLL: 50 ± 7%; dVNLL: 32 ± 5%, *p < 0.05, unpaired student’s *t*-test). This is a further indication that excitatory inputs differ between the ventrolateral region and the other regions of the VNLL, whereas inhibitory input properties are very similar amongst neurons in the entire VNLL.

### Neurons in the Dorsal and the Ventral VNLL are all Inhibitory

**Figure 7:**
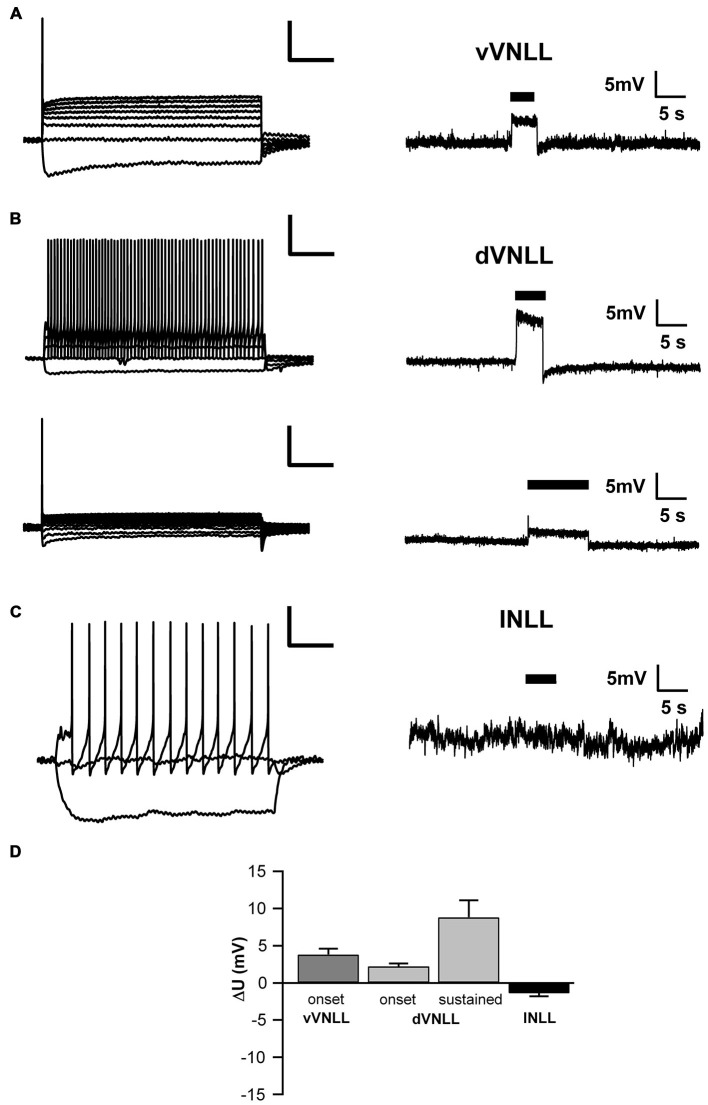
**Both vVNLL and dVNLL neurons are inhibitory. (A)** Representative voltage trace of a vVNLL neuron to light stimulation (indicated by the black bar). The neuron depolarizes in response to the light pulse. The left panel depicts the corresponding firing pattern of the neuron. **(B)** Representative voltage traces of an onset-type and a sustained firing dVNLL neuron to light stimulation (indicated by the black bar). Both neurons depolarize in response to the light pulse. The left panels illustrate the corresponding firing patterns of the respective dVNLL neuron. **(C)** Representative voltage trace of an INLL neuron to light stimulation (indicated by the black bar). The left panel depicts a representative IV curve for INLL neurons. **(D)** Voltage deflection in response to light stimulation in VNLL and INLL neurons (vVNLL: *n* = 6, dVNLL: sustained *n* = 6, onset-type *n* = 9; INLL: *n* = 8). Data are obtained from seven mice. Scale bar insets: 200 ms, 20 mV.

To test whether the neurons we recorded from were inhibitory and therefore part of the VNLL, we recorded from neurons of VGAT-ChR2-YFP^+^ mice. We tested the transmitter phenotype of neurons in both the vVNLL and dVNLL by applying short pulses of white light onto the slice while recording the membrane potential. All VNLL neurons, in the dorsal and ventral part, showed a pronounced depolarization in response to the light stimulus (Figures 7A,B,D). The amplitude of the depolarization varied dependent on the input resistance of the neuron (vVNLL: 3.8 ± 0.8 mV, *n* = 6; dVNLL: onset-type 2.2 ± 0.4 mV, *n* = 9, sustained 8.8 ± 2.3 mV, *n* = 6; INLL: −1.4 ± 0.4 mV, *n* = 8; Figure 7D). A population of small neurons in between our region of interest (dVNLL) and the DNLL did not depolarize during light application (Figures 7C,D). We therefore consider this population as glutamatergic and according to Ito et al. (2011) as INLL neurons, whereas our sample of neurons show an inhibitory neurotransmitter type and are thus located within the VNLL.

## Discussion

The present study demonstrates several distinct subpopulations of neurons in the dorsal and ventral parts of the VNLL. One distinct subpopulation of neurons, located in the ventrolateral part of the VNLL, displayed an onset-type firing pattern and only little I_h_. These neurons received mostly one large calyx-like excitatory input, multiple large inhibitory inputs and were densely packed in a cluster lateral to the fiber bundle. A second cell type, located in the ventromedial part of the VNLL, was scattered within the fiber bundle. Although these neurons exhibited similar firing and intrinsic properties compared to ventrolateral neurons, they received several and much weaker excitatory inputs. In the dVNLL, neurons with two different firing patterns are intermingled. Neurons showed either a single spike at the onset of the response or a sustained firing pattern. Moreover, all dorsal neurons exhibited large I_h_. The onset-type firing neurons in the dorsal part clearly differed from the onset-type firing neurons in the ventral part of the VNLL in terms of their intrinsic properties. Ventromedial VNLL neurons that were intermingled within the lemniscal fiber tract had intrinsic properties similar to the ventrolateral neurons and synaptic properties that resembled those of neurons in the dVNLL. All neuron types in the dorsal and ventral VNLL depolarized by light stimulation in a VGAT-Channelrhodopsin mouse model where channelrhodopsin is expressed under the promoter of VGAT (Zhao et al., 2011). Since in this mouse model only inhibitory neurons depolarize by light stimulation our sample neurons were inhibitory and were rather part of the VNLL and not the INLL (Saint Marie et al., 1997; Ito et al., 2011).

### The Ventral Part of the VNLL Contains a Cluster of Neurons with Calyx-Like Synaptic Inputs

This study describes a distinct population of neurons in the vVNLL with biophysical membrane properties that are very similar to those found in neurons of the MNTB (Brew and Forsythe, 1995; Hassfurth et al., 2009). Both of these neuron populations have a typical onset-type response pattern, little I_h_ and a lack of HCN1 immunoreactivity. Previously, similar onset-type firing neurons have been described in the ventral part of the rat VNLL which were correlated with a bushy-cell like morphology (Wu, 1999; Zhao and Wu, 2001). However, only around 25% of the neurons studied belonged to this neuron-type. Explanation for this relatively low fraction of onset neurons might be the different developmental time frames studied, variations in recording sites (dorsal vs. ventral) and the different recording techniques employed. A very recent study of gerbil VNLL neurons with a Calyx-like input demonstrated a developmental change from a sustained to an onset-type firing pattern within the first week after hearing onset (Berger et al., 2014; Franzen et al., 2015). However, neither the spatial distribution of neurons within the VNLL nor the existence of other cell types was discussed in this study.

Our data show that these neurons receive one large calyx-like excitatory input similar in amplitude and temporal properties to the Calyx of Held synapse in the MNTB (Borst and Soria van Hoeve, 2012). Moreover, these neurons are surrounded by a VGluT1 and calretinin positive ring, which suggests that this input originates from octopus cells of the contralateral VCN (Adams, 1997; Schofield and Cant, 1997). Similar calyx-like synaptic structures with comparable physiological characteristics were recently described in the gerbil VNLL (Berger et al., 2014). However, in this study VNLL neurons were usually contacted by two large excitatory synaptic inputs. Whether this is a species specific adaptation or whether a further developmental reduction of inputs occurs after P12, when these experiments were performed, remains unexplored. Anatomical studies in other mammals identified a subdivision within the ventral VNLL with one densely and fairly uniform arrangement of neurons with spherical or globular bushy cell morphology (Willard and Ryugo, 1983; Vater and Feng, 1990; Adams, 1997; Schofield and Cant, 1997; Benson and Cant, 2008), which suggests that this neuron type is commonly found in all mammalian species. In fact, an anatomical study suggests that in humans this area is even hypertrophied (Adams, 1997). Interestingly, neurons in the ventral VNLL, that were not located in this lateral cluster but were intermingled within the fiber tract of the lateral lemniscus, received several excitatory inputs with much smaller amplitude similar to dVNLL neurons, while intrinsic properties were indistinguishable from the ventrolateral VNLL neurons. Further experiments are needed to determine whether input origin and response properties of these neurons are more similar to ventrolateral or dorsal VNLL neurons.

### Neurons in the Dorsal Part of the VNLL have Two different Firing Patterns and Large I_h_

Dorsal VNLL neurons responded with two different firing patterns to depolarizing current injections. Whereas one population of neurons had the typical onset-type response similar to principal cells in the lateral and medial superior olive with low input resistance and large I_h_ (Mathews et al., 2010; Walcher et al., 2011), the other neuronal population fired throughout the depolarizing stimulus and with increasing frequency for stronger depolarization. This suggests a differential distribution of K-channels, especially the low-threshold K-channels of the Kv1 family, that are usually abundant in neurons with an onset-type firing pattern (Barnes-Davies et al., 2004; Mathews et al., 2010). Morphologically, two different neuron types are present in the dVNLL. The most frequent one are multipolar cells that are densely packed in the most dorsal part of the VNLL in most species (Schofield and Cant, 1997; Benson and Cant, 2008). Another less frequent type are the giant cells (Schofield and Cant, 1997; Benson and Cant, 2008). Whether this morphological cell type corresponds to our onset or sustained firing type is unclear, but could be determined by studying K-channel distribution in the VNLL.

In the dVNLL all neurons had relatively large I_h_ by visually identifying groups and displayed intense immunostaining against the HCN1 channel. Among other HCN subtypes the HCN1 subunit is the one with the fastest activation time constant and the most depolarized half-maximal activation of I_h_ (Wahl-Schott and Biel, 2009). Therefore, neurons with large HCN1 mediated currents integrate excitatory and inhibitory inputs with a high temporal resolution and little summation (Khurana et al., 2012; Baumann et al., 2013). Consistently, most of the neurons received excitatory and inhibitory inputs with fast synaptic time constants. These fast synaptic currents together with the rapid membrane time constants suggest that these neurons might contribute to temporal sound pattern analysis by integrating their inputs with high temporal precision as suggested for VNLL neurons (Nayagam et al., 2005; Yavuzoglu et al., 2011; Recio-Spinoso and Joris, 2014).

### Dorsal and Ventral VNLL Neurons are Inhibitory

Several studies have shown that a population of neurons in the most dorsal VNLL differs in terms of input projection, binaurality and neurotransmitter type compared to more ventral VNLL neurons. These dorsal VNLL neurons mostly express excitatory markers whereas most neurons in ventral VNLL are either GABAergic, glycinergic or co-express both inhibitory transmitters (Saint Marie et al., 1997; Riquelme et al., 2001; Ito et al., 2011). The very dorsal VNLL neurons also receive prominent projections from binaural nuclei of the superior olive (Kelly et al., 2009) which are mostly absent in the ventral VNLL. A number of studies have therefore defined this structure in the very dorsal VNLL as a distinct nucleus, named the intermediate nucleus of the lateral lemniscus (INLL). Nevertheless, the borders and the extent of this structure are not clearly defined and show large species specific differences (Zook and Casseday, 1982; Saint Marie et al., 1997; Kelly et al., 2009; Ito et al., 2011). Using a transgenic mouse model, which expresses channelrhodopsin-2 under the VGAT promoter and thus only in inhibitory, GABAergic or glycinergic neurons (Zhao et al., 2011), we show that all our neurons in the dorsal VNLL are inhibitory and therefore presumably not part of the excitatory, binaural INLL structure. We also identified a population of small neurons that are located dorsal of our investigated neurons might fulfill this criteria.

### Functional Heterogeneity of VNLL Neurons for Sound Processing

The present study shows distinct variations in intrinsic and synaptic properties between the different population in the ventral and dorsal VNLL. *In vivo* several response patterns to sounds including onset, regular, sustained and chopper cells have been recorded in several species, however, with no clear correlation between response type and location within the VNLL (Batra and Fitzpatrick, 1997; Nayagam et al., 2006; Zhang and Kelly, 2006). One possible explanation for this discrepancy is that the *in vivo* response patterns are mainly determined by the input properties whereas the intrinsic and synaptic properties are less influential. A similar lack of position with binaural response type of VNLL neurons was observed in most species examined. In the rat and cat most VNLL neurons are driven by contralateral stimulation with more pronounced ipsilateral inhibitory inputs in the rat but only weak influence by ipsilateral stimulation in the cat (Nayagam et al., 2006; Recio-Spinoso and Joris, 2014; Zhang and Kelly, 2006). A similar weak input from the ipsilateral ear is present in the mouse VNLL (unpublished observation). It is therefore unlikely that the two different response types in the dVNLL correspond to different groups of binaurality. We observed inhibitory, glycinergic inputs with similar characteristics to all neuron types and in all regions of the VNLL. Whether these inputs all derive from the previously characterized input of the ipsilateral MNTB (Kelly et al., 2009) or whether some of these inputs originate from other glycinergic nuclei, as e.g., the contralateral MNTB or the lateral nucleus of the trapezoid body, needs to be shown.

Finally, many anatomical and physiological observations suggest that the VNLL is involved in various forms of temporal processing of sound patterns (Covey and Casseday, 1991; Adams, 1997; Oertel, 1999; Nayagam et al., 2005). Especially the bushy cell-like neurons in the vVNLL that receive calyx-like inputs have received attention in this context. In the mouse we have identified a cluster of neurons in the ventrolateral part of the VNLL that receives a single calyx-like input that most likely originates from the octopus cells in the PVCN. Octopus cells preferably respond to broadband stimuli with precisely timed discharges (Rhode et al., 1983; Rouiller and Ryugo, 1984). Similarly, VNLLc neurons in bats respond with exceptionally sharply timed spikes to the onset of sounds (Covey and Casseday, 1991). Likewise, a population of VNLL neurons in other mammals shows precisely timed onset responses (Nayagam et al., 2005; Recio-Spinoso and Joris, 2014). These neurons are monaural and respond with one spike with short latencies precisely locked to the stimulus onset which makes these neurons suited to accurately encode temporal information (Recio-Spinoso and Joris, 2014). In accordance with this, ventrolateral VNLL neurons exhibit intrinsic properties that secure temporally precise information processing (Franzen et al., 2015). A recent model suggests that these neurons provide fast onset inhibition to neurons in other parts of the VNLL and the IC thereby suppressing spectral splatter (Spencer et al., 2015). Indeed, a previous study illustrates that a subpopulation of VNLL neurons receives a large preceding inhibition which strongly influences first spike latency (Nayagam et al., 2005; Spencer et al., 2015). The fast membrane and synaptic properties of the onset-type neurons of the dVNLL would be ideally suited to precisely integrate the fast preceding inhibition with several excitatory inputs. Nevertheless, in none of our recordings from dVNLL neurons we were able to detect an extremely large inhibitory input, as proposed in the model study (Spencer et al., 2015). It is possible that these large inhibitory inputs are received by a special type of neuron that is not preserved in the brain slice preparation. Alternatively, our stimulation method could be less effective in stimulating these inputs. Using the VGAT-channelrhodopsin mouse model could provide more information about this intrinsic inhibitory circuit.

## Conclusion

We found distinct populations of neurons with different biophysical and synaptic properties in the dorsal and ventral parts of the VNLL. One neuron type in the ventrolateral VNLL displays onset-type response pattern and calyx-like excitatory input properties very similar to MNTB neurons. These properties makes these neurons likely candidates for providing temporally precise and fast feed-forward inhibition to target cells within the VNLL and to the IC. In contrast onset-type dVNLL neurons with large I_h_ and short membrane time constants might contribute to the analysis of temporal sound pattern by integrating excitatory and inhibitory inputs on a fast time scale.

## Author Contributions

VB, FC and UK conception and design of research; VB, FC and EG performed experiments; VB, FC and UK analyzed data; VB, FC and UK interpreted results of experiments; VB, FC and EG prepared figures; VB, FC, EG and UK drafted and revised manuscript, and approved final version of manuscript.

## Funding

This work was supported by Deutsche Forschungsgemeinschaft (SFB 665, SPP1608) and NeuroCure.

## Conflict of Interest Statement

The authors declare that the research was conducted in the absence of any commercial or financial relationships that could be construed as a potential conflict of interest.
